# Non-Mammalian Vertebrates: Distinct Models to Assess the Role of Ion Gradients in Energy Expenditure

**DOI:** 10.3389/fendo.2017.00224

**Published:** 2017-09-01

**Authors:** Caroline E. Geisler, Kyle P. Kentch, Benjamin J. Renquist

**Affiliations:** ^1^School of Animal and Comparative Biomedical Science, University of Arizona, Tucson, AZ, United States

**Keywords:** ectotherm, endotherm, energy expenditure, membrane potential, mitochondrial membrane potential, H^+^ gradient, Ca^++^ gradient, Na^+^/K^+^ gradient

## Abstract

Animals store metabolic energy as electrochemical gradients. At least 50% of mammalian energy is expended to maintain electrochemical gradients across the inner mitochondrial membrane (H^+^), the sarcoplasmic reticulum (Ca^++^), and the plasma membrane (Na^+^/K^+^). The potential energy of these gradients can be used to perform work (e.g., transport molecules, stimulate contraction, and release hormones) or can be released as heat. Because ectothermic species adapt their body temperature to the environment, they are not constrained by energetic demands that are required to maintain a constant body temperature. In fact, ectothermic species expend seven to eight times less energy than similarly sized homeotherms. Accordingly, ectotherms adopt low metabolic rates to survive cold, hypoxia, and extreme bouts of fasting that would result in energy wasting, lactic acidosis and apoptosis, or starvation in homeotherms, respectively. Ectotherms have also evolved unique applications of ion gradients to allow for localized endothermy. Endothermic avian species, which lack brown adipose tissue, have been integral in assessing the role of H^+^ and Ca^++^ cycling in skeletal muscle thermogenesis. Accordingly, the diversity of non-mammalian vertebrate species allows them to serve as unique models to better understand the role of ion gradients in heat production, metabolic flux, and adaptation to stressors, including obesity, starvation, cold, and hypoxia.

## Introduction

The ease of genetic manipulation, the standard husbandry demands, and the well-established experimental methods have resulted in rodent model predominating the obesity field. Yet, the diversity of non-mammalian vertebrates, including both endothermic (birds and some fish) and ectothermic (amphibians, reptiles, and fish) species that have adapted to a variety of environments, provides unique opportunities to better understand the regulation of energy expenditure. The heterogeneity of species and environments to which they have adapted yield an abundance of unique attributes that can better help us understand energy expenditure. Herein, we review research in non-mammalian vertebrate control of electrochemical gradients and application of this work to mammalian species.

Basal metabolic rate is seven- to eightfold higher in mammals than in similarly sized ectotherms maintained at 37°C (1, 2). Because ectothermic species have low levels of basal heat production, they are sensitive models to identify the role of ion flux in altering heat production and basal metabolic rate. For example, egg brooding female pythons use whole body skeletal muscle contractions to facilitate endothermy and maintain a body temperature 9–13°C above ambient temperature (3, 4). This contraction-induced thermogenesis increases the oxygen consumption 22 times above baseline (5). In rat pups (22 days), maintenance of body temperature in an environment 9–13°C, colder than thermoneutral, increases oxygen consumption just two times above baseline (6). While in penguin chicks, a temperature drop of 10°C below the shivering threshold only increases oxygen consumption by 27%, while a temperature 25°C below the shivering threshold doubles oxygen consumption (7). Thus, the low basal metabolic rate of ectothermic vertebrate species allow for a uniquely sensitive model to perturbations in metabolic rate, while the endothermic bird provides a comparative model to better understand perturbations and adaptations that affect whole body and skeletal muscle energy expenditure in the absence of endothermic brown adipose tissue.

Herein, we review the lower vertebrate literature on energy expenditure with a focus on three ion gradients: the H^+^ gradient at the inner mitochondrial membrane, the Ca^++^ gradient of the sarcoplasmic reticulum, and the Na^+^/K^+^ gradient across the plasma membrane. We further apply findings from the lower vertebrate literature to our current understanding of mammalian energy expenditure and its potential application to human health.

## H^+^ Gradient

**Figure 1:**
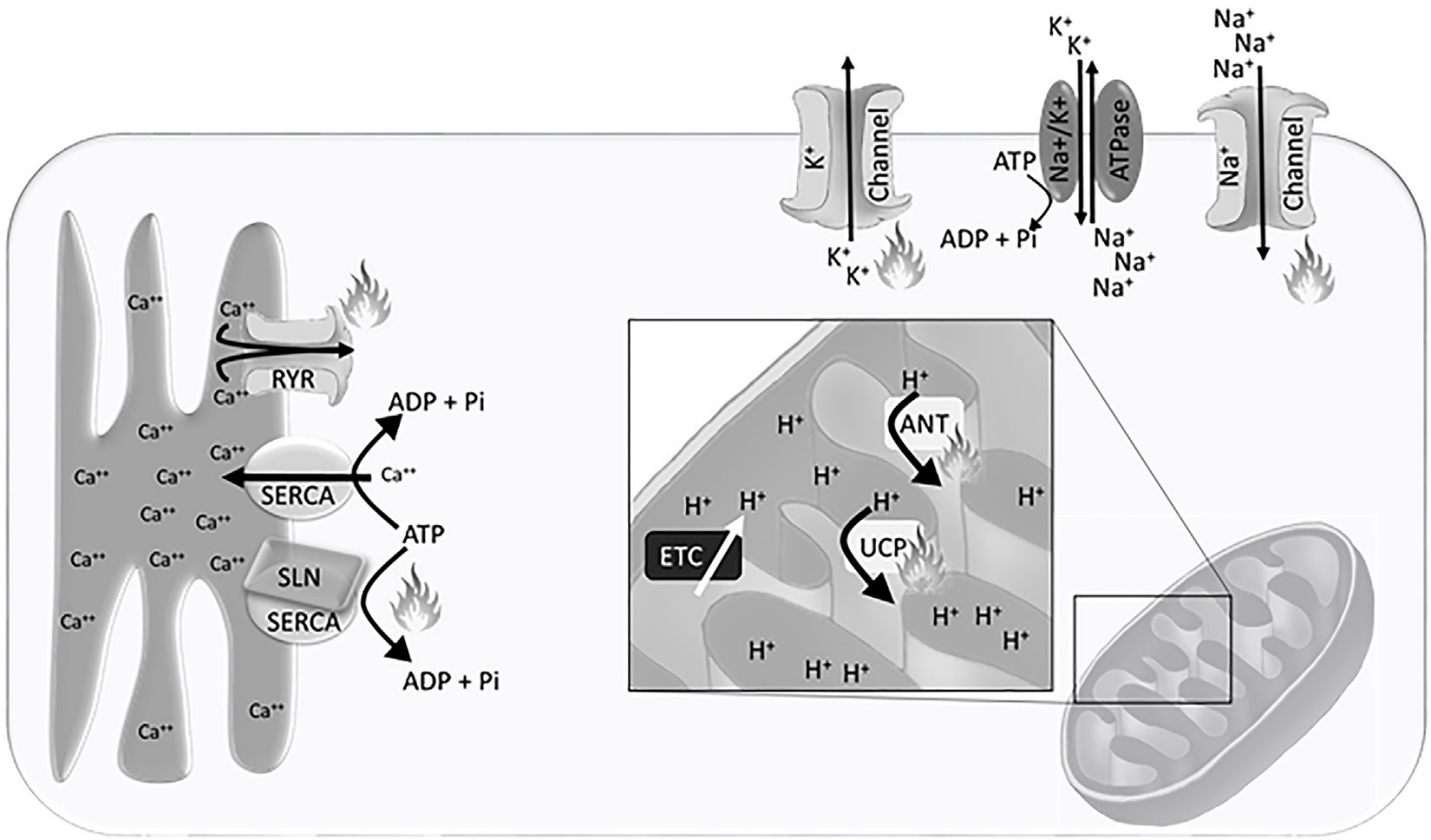
Schematic representation of the Ca^++^ gradient at the sarcoplasmic reticulum, H^+^ gradient at the inner mitochondrial membrane, and Na^+^/K^+^ gradient at the plasma membrane. Flames indicate sites of heat generation from ion leak and non-functional adenosine triphosphate (ATP) hydrolysis. Abbreviations: SERCA, sarco/endoplasmic reticulum Ca^++^ ATPase; RYR, ryanodine receptor; SLN, sarcolipin; ETC, electron transport chain; UCP, uncoupling protein; ANT, adenine nucleotide translocase.

The H^+^ gradient is maintained in the inter-mitochondrial membrane space, created by electron transport chain activity (Figure 1). This gradient is dissipated by H^+^ ion leak across the inner mitochondrial membrane, which can be exacerbated by uncoupling proteins (UCP) and adenine nucleotide translocases (ANT), or production of adenosine triphosphate (ATP) through ATP synthase. Accordingly, the mitochondrial density, inner-mitochondrial membrane surface area, expression and activity of electron transport chain proteins, and the expression of UCPs and ANTs that allow for H^+^ leak across the inner mitochondrial membrane are integral to heat production and energy expenditure.

Compared to endotherms, reptilian tissues have fewer mitochondria with less inner mitochondrial membrane surface area per mitochondria, resulting in 50% less inner mitochondrial membrane across which H^+^ can leak (1). Because the organs that are rich in mitochondria are smaller in ectotherms, whole body mitochondrial membrane surface area is four times greater in mammals than in reptiles of the same body size (8). The H^+^ gradient is established by electron transport chain activity and depressed by the release of H^+^ out of the inter-mitochondrial membrane space. With four times less membrane surface area, ectotherms have lower electron transport chain activity and H^+^ ion leak.

### Electron Transport Chain

The decreased electron transport chain activity in ectotherms is not a result of decreased activity of proteins integral to oxidative phosphorylation (2, 9). ATP synthesis, expressed as a percentage of mitochondrial respiration, is similar in reptiles and mammals at similar body temperature (2). Inner mitochondrial membrane cytochrome *C* oxidase (complex IV) content and activity is also similar in fish and cattle (9). Moreover, when corrected for tissue protein content, cytochrome *C* oxidase activity is similar in reptiles and mammals (10). Thus, the decreased electron transport chain activity in ectotherms is a result of decreased mitochondria numbers, mitochondrial size, mitochondrial membrane area, and a resulting decrease in total electron transport proteins rather than altered activity of the mitochondrial machinery.

The electron transport chain represents an important point of manipulation for energy expenditure in ectotherms. During hypoxia and cold exposure, electron transport chain activity can be depressed to accommodate the decreased cellular energy demand imposed by these conditions. Thus, in species not dependent on internal heat production, limiting cellular respiration is an adaptive response to environmental stress. In fact, ectothermy allows for adaptive whole body cooling and metabolic depression to prevent metabolic acidosis in response to hypoxia (11). Four months of *in vivo* hypoxia results in a severe decrease in adenosine diphosphate (ADP) stimulated respiration (State 3) and a decrease in ADP-independent respiration (State 4; H^+^ leak) from isolated skeletal muscle mitochondria (12). Hypoxia induces a characteristic rise in plasma lactate in frogs maintained at 7°C, while there is no change in plasma lactate in frogs maintained at 1.5°C (13). The rise in lactate at 7°C is a result of glycolytic flux exceeding the mitochondrial capacity for oxidation under hypoxic conditions. In turn, frogs or toads challenged with hypoxia chose to reside at lower temperatures, decrease their metabolic rate, and limit the metabolic disturbances associated with a lack of oxygen (13–15). In alligators, hypoxia leads to hypothermia in an adaptive effort to depress metabolic demands (16). Long-term exposure to hypoxia and 3°C water results in an adaptive decrease in skeletal muscle mitochondrial oxygen consumption by inhibiting flux through the electron transport chain and limiting H^+^ ion leak across the inner mitochondrial membrane (12, 17–19). Indeed, ectothermic species exploit the decrease in metabolic demand that accompanies low body temperatures to adapt to acute or chronic hypoxic environments (14).

Chronic hypoxia, due to a high altitude habitat, also regulates mitochondrial respiration. In a comparison of two closely related lizard species that live at different elevations, species adapted to high elevations had lower oxidative capacity and less ADP-independent respiration than species that inhabit lower elevations (20). Thus, adaptation to chronic hypoxia increased the efficiency of H^+^ ion gradient energy capture as ATP. Of note, mitochondria from lizards adapted to high elevations are less able to increase respiration in response to an increase in incubation temperature (20). Suggesting that there is an adaptive cost to the tight coupling between the H^+^ ion gradient and ATP synthesis.

In endotherms, cold exposure increases electron transport chain activity to promote endogenous heat production. Acute, 24 h, cold exposure in the chick increases expression of cytochrome *C* oxidase and NADH ubiquinone oxidoreductase (complex I) (21). Chronic cold exposure similarly increases cytochrome *C* oxidase activity as a result of increased mitochondria number and increased inner mitochondrial membrane area/mitochondrial volume (22). In small mammals, the primary thermogenic organ is brown adipose tissue. Chronic, 4 week, cold exposure in the tree shrew increases the cytochrome oxidase activity in brown adipose tissue to six times that observed in thermoneutral conditions (23). The same 4-week cold exposure increases liver cytochrome oxidase activity 2.7 times (23). In mice, skeletal muscle electron transport activity is increased by cold acclimation (24). Mice that lack UCP1 and the thermogenic potential of brown adipose tissue, display an increase in skeletal muscle mitochondria to accommodate an increased thermogenic role for skeletal muscle (24). Together these studies suggest that cold exposure increases electron transport chain activity and mitochondrial density similarly in endothermic avian and mammalian systems. The necessity to use a UCP1 knockout mouse to better assess the role of skeletal muscle in thermogenesis recommends that birds, which lack brown adipose tissue, may be better suited models to understand the role of skeletal muscle thermogenesis in response to environmental or dietary stimuli.

### H^+^ Leak

H^+^ leak across the inner mitochondrial membrane uncouples the electron transport chain from oxidative phosphorylation, preventing the capture of energy as ATP and enhancing its release as heat. In rodent-based studies, heat production through H^+^ leak is primarily associated with the inducible thermogenesis observed in brown adipose tissue. However, the leak of H^+^ in liver and skeletal muscle mitochondria has been estimated to be responsible for 20% of basal energy expenditure in the rat (25). In fact, it accounts for 50% of respiration in perfused rat skeletal muscle (25). By comparing the respiration rate of mitochondria collected from rat and frog heart in the absence of ADP, Akhmerov showed that H^+^ ion leak was six- to sevenfold greater in isolated rat mitochondria than in isolated frog mitochondria (26). This may be in part explained by the inner mitochondrial membrane surface area per mitochondria being larger in mammals than in reptiles of a similar size (1). However, it may also be due to differences in the H^+^ gradient and membrane lipid composition.

Proton leak increases with mitochondrial membrane potential across species. Still, at the same mitochondrial membrane potential, H^+^ ion leak is approximately fivefold greater in the rat than in the bearded dragon (2). Other ectothermic species mute H^+^ leak by maintaining a lower mitochondrial membrane potential. In the rainbow trout, mitochondrial proton leak at a given membrane potential is higher than that observed in mitochondria from rat or pigeon (27). This is because membranes in cold-water fish species have a high degree of polyunsaturated fatty acyl groups to maintain membrane fluidity at low temperatures. The increase in polyunsaturation also increases ion permeability (28). To limit proton leak, the rainbow trout has low electron transport chain activity eliciting a low mitochondrial membrane potential. Accordingly, total proton leak is lower in the rainbow trout than in the endothermic rat and pigeon. Thus, proton leak can be manipulated by either altering electron transport chain activity and mitochondrial membrane potential or by changing H^+^ ion permeability.

Although proton leak is typically associated with heat production, increasing proton leak can also allow for metabolic flexibility. Accordingly, fish express UCP1, 2, and 3 (29, 30). In the carp, UCP1 is primarily expressed in the liver and expression is downregulated by a decrease in water temperature (30). The liver plays a central role in synthesizing and distributing glucose, ketones, and lipids to the rest of the body. Therefore, an increase in water and body temperature would increase the metabolic demand of non-hepatic tissues and necessitate expression of UCP1 to regenerate FAD^+^ and NAD^+^. In line with increased gluconeogenic demand, hepatic phosphoenolpyruvate carboxykinase (PEPCK) enzyme activity, essential for greater gluconeogenic flux, is increased in the Antarctic eelpout in response to a 5°C increase in water temperature (31). Thus, the direct relationship between temperature and UCP1 expression may encourage the regeneration of hepatic FAD^+^ and NAD^+^, essential for flux through β-oxidation to produce the acetyl-CoA necessary for ketogenesis while sparing amino acid oxidation. Accordingly, in the fish upregulation of UCP1 is not an adaptation to increase hepatic heat production, but instead to promote metabolic flexibility. Skeletal muscle primarily expresses UCP3 in both fish and rodents (30, 32). Interestingly, UCP3 is also similarly upregulated by fasting in both rodents and fish, while fasting increases proton leak from skeletal muscle mitochondria in the cane toad (15). As we proposed for UCP1 in liver, it has been proposed that this upregulation of UCP3-mediated H^+^ leak is essential for the increased reliance on fatty acid oxidation during a fast (32). Thus, studies of UCP regulation in fish can improve our understanding of UCP-mediated changes in metabolic flux without the demand for heat generation.

Conversely, some ectothermic fish species appear to manipulate H^+^ leak to warm-specific tissues above environmental temperature. Indeed, cold acclimation increases UCP1 expression in the optic tectum, a brain region important for sight, in the carp (33). This warms the brain’s optic center to maintain eyesight in cold environments, through a mechanism similar to the Ca^++^ cycling-based heater organ in deep diving marine species (34, 35). In sharks, skeletal muscle endothermy is thought to increase swimming rate, allow for thermal niche expansion, and increase digestive enzyme activity and digestive system passage (36). The mako shark uses red muscle fibers for endothermia. Mitochondria from mako shark red muscle fibers and liver display increased succinate stimulated respiration and maintain membrane potentials that exceed that of mitochondria from two ectothermic sharks. Although mitochondria from all of these shark species have similar levels of proton leak at a given membrane potential, the increased proton gradient in mako sharks drives H^+^ leak and heat production (37). Together these studies suggest that heat production through H^+^ leak may be used in some ectothermic species to warm specific tissues or maintain a set minimal body temperature required for survival.

In endotherms (birds and mammals), proton leak decreases with increasing body mass and explains 67% of the variability in standard metabolic rate (27, 38, 39). Proton leak is robust in birds. In fact, liver mitochondrial proton leak in all birds studied, including the much larger emu and goose, exceeded that of the rat (39). Across bird species, the ratio of monounsaturated fatty acids in phospholipids increases with body mass (39). The increase in monounsaturated fatty acyl groups may play a role in the decreased proton leak associated with increased body mass. In fact, membranes rich in monounsaturated fatty acyl groups are typical in ectothermic species with low levels of membrane ion permeability (40).

In rodents, studies aimed at understanding the endothermic response to cold have focused on the role of UCP1-mediated H^+^ ion leak in brown adipose tissue (41). Brown adipose tissue is integral to the maintenance of homeothermy in newborn infants (42–44). However, in humans, the passage from newborn through childhood and into adulthood results in a decrease in the amount of UCP in adipose tissue, suggestive of a reduction in metabolically active brown adipose tissue (45). The recent identification of brown adipose tissue depots near the clavicle in the adult human has spurred a resurgence in research aimed at better understanding the role of brown adipose tissue in energy expenditure of adult humans (46, 47). Yet the relative mass of brown adipose tissue in the adult human is minimal (168 ± 56 cm^3^). Accordingly, animal models that lack brown adipose tissue may provide a better understanding of the role of H^+^ leak in the thermogenesis of other tissues (48).

Birds, which lack brown adipose tissue, are uniquely suited to address the role of H^+^ leak in the endothermic response to environmental stimuli (cold or fasting). Avian UCP is expressed in skeletal muscle and increases in cold acclimated ducklings and penguins (49, 50). Proton leak across the inner mitochondrial membrane is higher in skeletal muscle from penguins that have been exposed repeatedly to cold water over 20 days. Teulier et al. observed that cold exposure of ducklings increased whole animal metabolic rate and ADP-stimulated oxidative phosphorylation. Surprisingly, despite the upregulation of avian UCP expression, there was no effect of cold acclimation on the efficiency of ATP synthesis or non-phosphorylating respiration. Accordingly, Teulier et al. propose that avian UCP primarily increases heat production by increasing aerobic skeletal muscle metabolic flux, rather than enhancing H^+^ leak (51).

In addition to the UCPs, the ANT may encourage proton leak through the inner mitochondrial membrane (52). Avian ANT protein expression increases in response to repeated cold water immersion (50). Therefore, both ANT and UCP are upregulated in cold acclimated birds and may be integral to the maintenance of body temperature through upregulated skeletal muscle thermogenesis. During a fast, the maintenance of body temperature may partially depend on mitochondrial H^+^ leak. Skeletal muscle avian UCP expression increases with glucagon treatment in ducklings (49). In the hummingbird, which undergoes torpor every night to endure an extended fast, skeletal muscle UCP expression increases during torpor and likely serves to help the bird re-warm from the torpid state (53). Thus, skeletal muscle is an integral site of mitochondrial proton leak, a key source of heat production in endotherms.

## Ca^++^ Cycling

Non-mammalian vertebrates have been integral to understanding the thermogenic potential of Ca^++^ cycling. Both fish and birds use Ca^++^ cycling to generate body heat independent of locomotion. To understand the unique physiological adaptations that each have evolved, we must first understand the basis of the Ca^++^ cycle. Within skeletal muscle the sarco/endoplasmic reticulum Ca^++^ ATPase (SERCA) pumps Ca^++^ from the cytosol into the sarcoplasmic reticulum against the concentration gradient, while hydrolyzing an ATP to ADP and inorganic phosphate. The hydrolysis of ATP releases some energy as heat. The remainder of the energy is released as heat when Ca^++^ leaks from the sarcoplasmic reticulum into the cytosol down the electrochemical gradient (Figure 1). This cycle is essential for calcium oscillations in muscle that allow for contraction.

Marlin, swordfish, and tuna all have an endothermic “heater” organ that raises the temperature of the brain and eye (35, 54–56). This heater organ is a highly vascularized, modified eye muscle rich in mitochondria and cytochrome oxidase activity (35, 54–56). By maintaining the central nervous system and eyes at temperatures above ambient temperature, the heater organ improves eye sight and central nervous system function in cold ambient temperatures (35, 56–58). Warming the retina improves temporal resolution up to 10-fold, giving these deep diving, visual predators a crucial advantage over prey species (56). In addition to being mitochondria rich (68% of total cell volume), the heater organ contains a great deal of sarcoplasmic reticulum rich in SERCA1b and the ryanodine receptor 1 (34, 55). This encourages heat production from ATP hydrolysis as the SERCA pumps Ca^++^ into the sarcoplasmic reticulum and additional heat production as that Ca^++^ flows through the ryanodine receptor 1 down the concentration gradient back into the cytosol (34). The lack of contractile proteins uniquely positions the heater organ to take full advantage of heat generation by calcium cycling without motor consequence (58).

Cold exposure increases basal metabolic rate in birds, rodents, and humans (59–62). However, unlike rodents, which rely heavily on brown adipose tissue for thermogenesis, birds lack brown adipose tissue and rely more heavily on skeletal muscle thermogenesis. In the Muscovy duck, *in vivo* cold exposure increases *ex vivo* skeletal muscle oxygen consumption by 25% (63). Acute cold exposure of ducklings increases cardiac output and directs blood flow toward thermogenic sites, resulting in a 130% increase in skeletal muscle blood flow (64), indicating that skeletal muscle is the primary thermogenic organ in the duckling. In fact, birds rely on skeletal muscle calcium cycling to maintain body temperature at low environmental temperatures. Accordingly, acute 24-h cold-exposure increases skeletal muscle Ca^++^ ATPase activity (21). Cold acclimation over 5 weeks in ducklings increases sarcoplasmic reticulum SERCA1, SERCA2, and ryanodine receptor expression and activity as evidenced by increased Ca^++^ uptake and ryanodine binding (65, 66). The increase in Ca^++^ cycling leads to an increased resting metabolic rate, ATP demand, and oxygen consumption in cold acclimated birds (63, 67, 68). This is accommodated by increased expression of components of the electron transport chain, including cytochrome *C* and NADH ubiquinone oxidoreductase (21).

Interestingly, cold exposure stimulates lipolysis in birds, rodents, and humans (21, 62, 69). Mobilized lipids provide a source of carbons to meet this increased metabolic demand associated with maintaining body temperature. Accordingly, in the mouse, fatty acid translocase (cd36) knockout prevents the maintenance of body temperature in response to cold exposure (70). Moreover, cold acclimation in the sparrow increases pectoralis cd36 protein expression by 46% (71). In addition to acting as a carbon source, these lipids may modulate the degree of Ca^++^ cycling. Long-chain acyl carnitines accumulate in the skeletal muscle of cold acclimated ducklings. Palmitoyl (16 C) carnitine activates Ca^++^ release from duckling skeletal muscle sarcoplasmic reticulum (72). Thus, this skeletal muscle non-esterified fatty acid accumulation and the potential downstream consequences are conserved from birds to rodents and humans (62).

Data from the partially endothermic fish species and homeothermic avian species has established the thermogenic potential of skeletal muscle and the role of skeletal muscle calcium cycling in heat generation and body temperature maintenance. More recently, studies focused on sarcolipin (SLN), a protein that uncouples ATP hydrolysis from sarcoplasmic reticulum Ca^++^ sequestration, have elucidated a mechanism by which mammals use this calcium sequestration machinery to generate heat (73). Normally, SERCA hydrolyzes ATP and uses the majority of that energy to pump Ca^++^ from the cytoplasm against a concentration gradient into the sarcoplasmic reticulum, while releasing the remaining energy as heat. By decreasing Ca^++^ sequestration, SLN increases the heat released per mol of ATP hydrolyzed by more than 50% (74–76). The Periasamy laboratory has conducted a set of elegant studies to investigate the role of SLN in the response to thermogenic and dietary challenges (77, 78). They first showed that UCP1 knockout mice, which lack the thermogenic potential of brown adipose tissue, express more SLN in skeletal muscle (78). SLN overexpression in skeletal muscle increases oxygen consumption (77). This increase in skeletal muscle oxygen consumption translates to whole body energy metabolism, as SLN overexpressing mice lose weight when pair fed with wild-type mice. Furthermore, when challenged with a high fat diet, these SLN overexpressing mice eat more yet gain less body weight than wild-type mice (77). These findings in the mouse may translate to humans, as obesity alters methylation in the promoter of the ryanodine receptor 1 gene (79). In fact, this effect on the RYR1 promoter is the most prominent methylation response to obesity, most heavily affected. Moreover, the normal pattern of methylation can be restored with weight loss (79).

Fish and birds have been essential in establishing the role of calcium cycling and the SERCAs in energy expenditure and heat production. Deep water fish have developed a unique tissue with specified function to maintain brain and eye temperatures during exposure to deep cold ocean waters, while endothermic birds establish the potential for skeletal muscle to act as a thermogenic organ. New research reporting that calcium cycling is important for body weight regulation in rodents provides an exciting new avenue for drug development to combat obesity. Humans and birds both express higher levels of SLN than mice, recommending that birds may be a valuable model organism for assessing the role of SLN in body weight regulation.

## Plasma Membrane Potential

The maintenance of the plasma membrane potential is a critical component of cellular homeostasis and requires the tight regulation of intracellular ion concentrations. Passive ion channels and active transport pumps establish Na^+^, K^+^, and Cl^−^ electrochemical gradients across the plasma membrane that are essential for fundamental cellular processes. Transport of molecules both into and out of the cell, hormone secretion, muscle contraction, and neuronal communication are all dependent on the maintenance of resting membrane potential and utilization of these energetically favorable ion gradients. The Na^+^/K^+^ ATPase is the primary active pump driving cellular membrane potential (Figure 1).

Na^+^/K^+^ ATPase activity represents a major cellular energy demand and significant portion of resting metabolic activity. At the whole animal level, the Na^+^/K^+^ ATPase accounts for ~25% of ATP consumption in mammals (80). However, this can vary widely by tissue. In the liver, Na^+^/K^+^ ATPase activity constitutes ~10% of cellular energy use, while this rises to 60% in brain and kidney (81). Comparing the relative contribution of energy consuming processes between endotherms and ectotherms, 60 and 54% of cellular respiration is directed toward ATP production, of which ~13.3 and ~18.5% is consumed by the Na^+^/K^+^ ATPase in rat and lizard hepatocytes, respectively (2, 82). Since the respiration rate of rat hepatocytes is about four times that of lizard hepatocytes, 5.6 times more ATP is allocated toward Na^+^/K^+^ ATPase activity.

While endotherms and ectotherms maintain similar Na^+^/K^+^ ATPase densities across tissue type, the molecular activity of the pump is four to five times higher in endotherms (83). Accordingly, the plasma membrane passive permeability to both Na^+^ and K^+^ is four- to ninefold greater in endotherms than in ectotherms at the same temperature (84–86). Thus, to maintain established ion gradients, the leakier cell membranes of endotherms require more active Na^+^/K^+^ ATPase. Increased total ion flux in endotherms may be an evolutionary adaptation to increase heat production. The resulting higher activity of the Na^+^/K^+^ ATPase, in part, accounts for the greater level of energy expenditure in endotherms.

The lipid composition of the plasma membrane has a primary role in regulating Na^+^/K^+^ ATPase activity. Among ectotherms, the degree of membrane polyunsaturation is significantly correlated with Na^+^/K^+^ ATPase activity (87). In membrane crossover experiments, reconstitution of the Na^+^/K^+^ ATPase of an ectotherm (cane toad or crocodile) in the more polyunsaturated membrane of an endotherm (rat or cattle) increases pump activity by 40–180% while reconstitution in the reverse direction decreases Na^+^/K^+^ ATPase activity by 40–250% (88, 89). Thus, the inherent properties of the plasma membrane strongly regulate Na^+^/K^+^ ATPase activity. Interestingly, membranes of cold water fish have higher levels of unsaturated phospholipids than their warm water counterparts (90). In carp liver slices, exposure to decreasing temperatures immediately inhibits the synthesis of saturated fatty acids and stimulates desaturase activity (91). Across fish species, acclimation to low temperatures increases the degree of membrane fatty acyl unsaturation, stimulating Na^+^/K^+^ ATPase activity to compensate for the cold-induced decline in enzyme activity (92). Ectotherms adapt to temperature by changing membrane composition, representing a key regulatory mechanism by which activity of membrane-integrated enzymes, including the Na^+^/K^+^ ATPase, are altered to maintain membrane potential in the face of variable environmental conditions. Still, temperature changes have a more robust effect on active pump processes than passive ion leak, giving rise to the possibility of temperature disrupted ion gradients. In fact, in skeletal muscle of cane toads, bullfrogs, and black racer snakes, intracellular Na^+^ concentrations are higher at 20°C compared to 30°C, suggestive of decreased Na^+^/K^+^ ATPase activity with increasing environmental temperature (93). In cold-adapted species, activity of the Na^+^/K^+^ ATPase is less temperature sensitive, limiting temperature-dependent changes in membrane potential. In fact, the molecular activity of the Na^+^/K^+^ ATPase from an Antarctic octopus is 400% greater than that of the temperate octopus at 10°C (94).

Many ectotherms that overwinter underground or are exposed to prolonged water submersion have developed strategies to tolerate hypoxia. Turtles can spend over half of their life in an overwintering state and survive sustained periods of anoxia (95). In the western painted turtle hepatocyte, 28% of total cellular ATP is utilized by the Na^+^/K^+^ ATPase in normoxia (96). In response to anoxia, the Na^+^/K^+^ ATPase activity decreases by 75%, but accounts for nearly three-fourth of total cellular ATP turnover. Suppressed Na^+^/K^+^ ATPase activity is partially mediated by adenosine signaling which is robustly stimulated during anoxia (97). Ion gradients and plasma membrane potential are maintained during anoxia. Thus, passive ion flux must be downregulated in anoxia to match the decrease in active ion transport. In the turtle, neurons adapt to low oxygen by limiting K^+^ leak through a fivefold reduction in the open probability of Ca^++^ gated K^+^ channels (98). However, not all ectotherms can maintain ion gradients when challenged with low oxygen. In rainbow trout hepatocytes, an anoxia-intolerant species, cellular ATP content, and Na^+^/K^+^ ATPase activity are rapidly reduced by anoxia while K^+^ efflux rates exceed K^+^ influx up to eightfold (99). In the anoxia-tolerant goldfish, anoxia diminishes K^+^ efflux to match the decline in Na^+^/K^+^ ATPase activity, resulting in a net flux of K^+^ close to 0. Therefore, downregulating ion leak and allocating a greater percentage of total cellular ATP to the Na^+^/K^+^ ATPase appear to be principal strategies in maintaining membrane potential during metabolic arrest in anoxia-tolerant species.

This comparison between anoxia tolerant and intolerant fish species recommends that hypoxia-induced cellular damage may be limited by inhibiting K^+^ efflux channels within cells. Indeed, hypoxia induces K^+^ efflux in mammalian tissues (100–102). In fact, inhibition of K^+^ efflux across the plasma membrane prevents neuronal apoptosis in rats subjected to transient middle cerebral artery occlusion to induce hypoxia and ischemia or in serum-starved apoptosis-induced mouse neocortical neurons (103). In addition, the role of K^+^ efflux in hypoxia-induced apoptosis may be used to better understand the resistance of cancer to hypoxia-induced apoptosis. Several human cancers express low levels of K^+^ channels, preventing K^+^ efflux, and resulting in resistance to apoptosis (104). As in the anoxia tolerant goldfish, this diminished K^+^ efflux, allows cancer cells to maintain a nearly normal membrane potential in the face of depressed Na^+^/K^+^ ATPase activity. Thus, the strategies developed by anoxia tolerant species may be used to limit hypoxia induced tissue damage caused by cardiac arrest or arterial occlusion, while the sensitivity of anoxia intolerant species may provide insight into treatments that will diminish the hypoxia resistance observed in cancer.

## Conclusion

Maintaining ion gradients demands at least 50% of mammalian resting metabolic energy expenditures. By adapting body temperature to the environment, ectotherms are not reliant on ion flux down the mitochondrial membrane H^+^, sarcoplasmic reticulum Ca^++^, or plasma membrane Na^+^/K^+^ gradients for internal heat generation. Because they are not as stringently dependent on the energetic costs of endogenous thermoregulation, ectotherms can manipulate these ion gradients to adapt to a wide range of environments and stressors. Like mammals, avian species are constrained by the demands of homeothermy. Yet, they lack the endothermic brown adipose tissue depots that constrain interpretation of rodent-based findings. In turn, having adapted to a range of environments, avian species provide an ideal comparative model to understand the regulation of ion flux to manipulate heat generation. The diversity of non-mammalian vertebrate species and environmental niches to which they have adapted provide unique insight into ion gradients that are lacking in studies of mammalian species. Ectothermic fish manipulate H^+^ and Ca^++^ leak to warm-specific tissues, providing ideal models to further investigate the regulation of ion leak. Avian species have been essential in establishing the importance of skeletal muscle mitochondrial density and Ca^++^ ATPase activity in thermogenesis. Recent work highlighting the role of SLN in mammalian body weight homeostasis validates the application of these avian studies to understand mammalian physiology. Finally, studies that compare the ability of anoxia tolerant and intolerant fish species to maintain plasma membrane potential may be applied to prevent the damage associated with stroke- or cardiac arrest-induced hypoxia and ischemia. In addition, these studies can be applied to exacerbate sensitivity to hypoxia in cancer. The vast number of lower vertebrate species and the varied environments to which they have adapted allow for unique research opportunities that can be exploited to expand our knowledge of mechanisms underlying metabolic flux, energy expenditure, and body weight regulation.

## Author Contributions

CG, KK, and BR all made substantial contributions to the conceptual framework of this review, participated in the drafting and revising of the manuscript, approved this final version for submission, and agreed to be accountable for all aspects of the work.

## Conflict of Interest Statement

The authors declare that the research was conducted in the absence of any commercial or financial relationships that could be construed as a potential conflict of interest.

## Abbreviations

UCP, uncoupling protein; ANT, adenine nucleotide translocase; NADH, nicotinamine adenine dinucleotide; ATP, adenosine triphosphate; ADP, adenosine diphosphate.
